# TaxaScope: a container-native, visualization-centric workstation for genome-based bacterial taxonomy

**DOI:** 10.3389/fmicb.2026.1809734

**Published:** 2026-05-29

**Authors:** Yuxin Peng, Yue Jiang, Yong Jae Lee, Ju Huck Lee, Cha Young Kim, Jiyoung Lee

**Affiliations:** 1 Korean Collection for Type Cultures (KCTC), Biological Resource Center, Korea Research Institute of Bioscience and Biotechnology, Jeongeup, Republic of Korea; 2 Third Institute of Oceanography, Ministry of Natural Resources, Xiamen, China; 3 KRIBB School of Biotechnology, University of Science and Technology (UST), Daejeon, Republic of Korea

**Keywords:** bacterial taxonomy, genome-based taxonomy, phylogenomic analysis, species delineation, containerization, graphical user interface, standardization, data visualization

## Abstract

**Introduction:**

Genome-based bacterial taxonomy requires standardized and reproducible analytical workflows for species delineation and phylogenomic placement; however, the practical deployment of these workflows remains a significant barrier for experimental biologists and clinical scientists. Widely adopted tools such as Prokka, antiSMASH, and PhyloPhlAn underpin key steps in genome annotation, functional characterization, and phylogenomic reconstruction, but their practical deployment in routine laboratory settings, especially on Windows based systems, remains non trivial due to complex software dependencies and command line centric workflows. Existing solutions, including cloud-based platforms (e.g., Galaxy and KBase) and commercial software suites (e.g., CLC Genomics Workbench), partially alleviate these challenges but may also involve considerations related to data-privacy concerns, upload latency, storage quotas, shared computing resources, and recurring licensing costs.

**Methods:**

To address these limitations, we introduce TaxaScope, a graphical-interface-driven desktop workstation designed to support reproducible, genome-based bacterial taxonomy by integrating a curated set of community-validated tools for genome quality assessment, annotation, phylogenomic inference, genome relatedness estimation, and functional profiling within a unified local graphical user interface (GUI). By leveraging Docker- and Podman-based containerization behind a user-friendly frontend, TaxaScope provides version-locked, standardized execution environments across computing platforms without requiring manual dependency management or prior Linux expertise.

**Result and discussion:**

We demonstrate the utility of TaxaScope through a comprehensive re-analysis of *Pseudomonas putida* KCTC 1751T, illustrating how standardized taxonomic workflows can be executed locally while automatically generating high-quality circular genome maps and interactive functional reports suitable for downstream interpretation and figure preparation directly from native tool outputs. Collectively, TaxaScope lowers the technical barrier to standardized and reproducible genome-based bacterial taxonomy by providing a private, locally controlled, containerized workflow that complements cloud-based and commercial infrastructures for routine taxonomic research. By providing a containerized and visualization-oriented desktop environment, TaxaScope facilitates the standardized execution of established genomic tools, thereby bridging the gap between complex bioinformatic workflows and consistent bacterial taxonomy.

## 1 Introduction

The genomic revolution has fundamentally reshaped the landscape of microbiological research. With the widespread adoption of high-throughput sequencing (HTS) technologies, whole-genome sequencing (WGS) has become a routine component of modern microbiology and is now central to genome-based bacterial taxonomy, where species delineation, phylogenomic placement, and standardized genome reporting increasingly rely on whole-genome data (Shendure and Ji, 2008; Whitman, 2015; Chun et al., 2018). As the cost of sequencing a bacterial genome has dropped below that of many comprehensive biochemical assays (Wetterstrand, 2013), the primary bottleneck in taxonomic and genomic studies has shifted from data generation to data analysis (Stephens et al., 2015). This transition has exposed a growing computational divide within the biological sciences (Markowetz, 2015). On one end are bioinformaticians proficient in command-line interfaces (CLI), Linux based environments, and scripting intensive workflows; on the other are experimental biologists and clinical scientists, many of whom are actively engaged in bacterial taxonomy and systematics, whose expertise lies in experimental design and biological interpretation but who often lack the specialized computational training required to navigate complex bioinformatics software ecosystems (Barone et al., 2017; Mangul et al., 2019). This divide is further exacerbated by the structure of the contemporary bioinformatics software landscape. Many widely cited and community validated tools that underpin genome based taxonomy, central to tasks such as genome quality assessment, annotation, and phylogenomic reconstruction, are predominantly developed for UNIX based operating systems (Linux and macOS; Cock et al., 2013). Representative examples include CheckM2 for genome quality evaluation (Chklovski et al., 2023), Prokka for genome annotation (Seemann, 2014), and PhyloPhlAn for phylogenomic inference (Asnicar et al., 2020).

These tools typically depend on complex and tightly coupled software stacks, requiring specific versions of programming languages (e.g., Python and Perl), auxiliary libraries (e.g., BioPerl), domain-specific software (e.g., HMMER and BLAST+), and low-level system libraries such as glibc (Kanwal et al., 2017; Grüning et al., 2018). As a result, deploying and maintaining genome based taxonomic pipelines on Windows based systems, which remain prevalent in biological laboratories, has historically been non trivial and frequently hindered by software incompatibilities commonly referred to as “dependency hell” (Boettiger, 2015). In practice, conflicts arising from a single library version can compromise entire analytical workflows, leading to inconsistent execution environments and discouraging broader adoption of genome-based taxonomy among non-specialist users (Sandve et al., 2013). Researchers seeking to overcome these challenges currently rely on a limited set of solutions, each associated with distinct trade-offs. Cloud-based platforms such as Galaxy and KBase provide graphical user interfaces (GUIs) that abstract command-line operations and lower the entry barrier for non-specialist users (Arkin et al., 2016; Afgan et al., 2018). However, reliance on remote infrastructure introduces additional constraints that are particularly problematic for taxonomic studies, including dependence on network bandwidth for uploading large genomic datasets, restrictive storage quotas, and queue-induced delays during periods of high user demand, which may postpone iterative and time-sensitive analyses. Beyond performance considerations, data governance and privacy represent critical concerns in genome-based taxonomy. Uploading clinical isolates, type strains, or proprietary collections to public or shared servers is frequently restricted by institutional review boards or data management policies, limiting the applicability of cloud-based solutions in clinical, industrial, and taxonomic research contexts (Erlich and Narayanan, 2014; Kaye et al., 2015). Alternatively, commercial software suites such as CLC Genomics Workbench and Geneious Prime provide polished, locally installed graphical user interfaces that run natively on Windows systems. While these platforms are well suited for a range of routine analyses, their use is frequently constrained by recurring licensing costs that may be prohibitive for smaller academic laboratories, teaching environments, or long-term taxonomic projects (Morin et al., 2012). Moreover, proprietary implementations may limit analytical transparency, as underlying algorithms and parameterization are often closed-source or subject to vendor-specific modifications, complicating independent verification and long-term workflow standardization in taxonomic research (Ince et al., 2012).

To address these challenges, we developed TaxaScope, a container-native and visualization-centric desktop workstation explicitly designed to support reproducible, genome-based bacterial taxonomy in wet-lab settings. Rather than reimplementing existing algorithms, TaxaScope functions as a modular wrapper system that orchestrates established, open-source, and community-validated bioinformatics tools within isolated, version-controlled execution environments, thereby lowering the technical barrier to entry while preserving analytical rigor and transparency. By leveraging daemonless, OCI-compliant containerization implemented via Podman, TaxaScope decouples taxonomic workflows from the host operating system. This architecture enables researchers working on Windows-based systems to execute the same tool versions and runtime environments as those deployed on Linux-based high-performance computing infrastructures, supporting standardized execution across platforms without requiring manual dependency management or Linux expertise. TaxaScope integrates a curated suite of tools that are central to genome-based taxonomy, including genome quality assessment (CheckM2 and BUSCO; Simão et al., 2015; Chklovski et al., 2023), genome annotation (Prokka; Seemann, 2014), phylogenomic and genome relatedness inference (PhyloPhlAn and pyANI/AAI; Pritchard et al., 2016; Asnicar et al., 2020; Kim et al., 2021), and functional profiling (antiSMASH and dbCAN; Blin et al., 2023; Zheng et al., 2023). Beyond tool execution, TaxaScope addresses the well-recognized “last-mile” challenge in taxonomic bioinformatics by transforming heterogeneous, text-based outputs into interpretable and 114 results suitable for downstream interpretation and figure preparation through an automated visualization engine, eliminating the need for custom scripting or external visualization software.

In this manuscript, we describe the design and implementation of TaxaScope, with particular emphasis on its container-native architecture, batch-processing framework, and integrated visualization workflow. TaxaScope was specifically designed for bacterial taxonomy and novel taxon characterization based on isolate genomes. The platform is evaluated through case study using the genome of *Pseudomonas putida* KCTC 1751^T^ to demonstrate workflow integration, standardized execution, and usability within a genome-based bacterial taxonomy workstation. The results show that TaxaScope enables experimental microbiologists to perform standardized, reproducible genome-based bacterial taxonomy locally and efficiently.

## 2 Materials and methods

### 2.1 System architecture and implementation of TaxaScope

**Figure 1:**
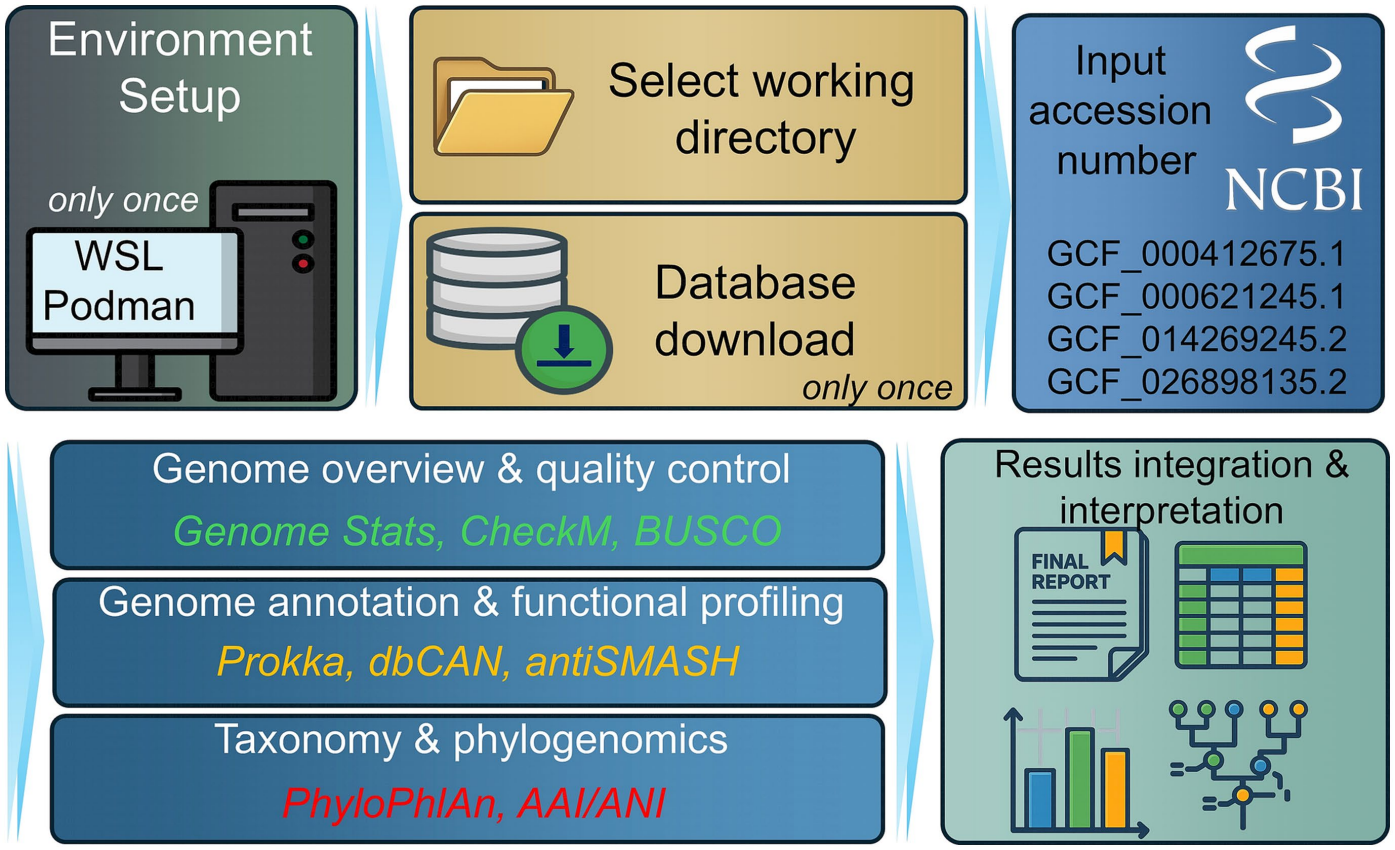
Overview of the TaxaScope workflow and functional modules. The workflow consists of three main stages. First, the environment is initialized (one-time setup) using WSL and Podman, followed by selection of the working directory and downloading of required databases. Genome inputs are provided via NCBI accession numbers. Second, genome analysis includes (i) genome overview and quality control (GenomeStats, CheckM, BUSCO), (ii) genome annotation and functional profiling (Prokka, dbCAN, antiSMASH), and (iii) taxonomy and phylogenomics (PhyloPhlAn, AAI/ANI). Third, results are integrated into a unified report for downstream interpretation. This modular design supports standardized execution of established genome-based analyses within a single desktop environment.

TaxaScope is a container-native desktop platform for standardized, genome-based bacterial taxonomy, built upon a modular architecture that separates user interaction from computational execution. As shown in Figure 1, the system is organized as a workflow-oriented pipeline, guiding users from environment setup and data input to sequential genome analysis, including quality control, annotation, phylogenomics, and functional profiling.

All analyses are executed within isolated container environments to ensure consistent tool behavior and standardized execution across systems. Outputs from individual modules are automatically integrated into structured reports and visualization results to facilitate downstream interpretation. Typical execution of TaxaScope requires at least 16 GB RAM and 50 GB of available storage, depending on the size of container images and associated reference databases.

### 2.2 Overall architectural design

The core design principle of TaxaScope is a strict decoupling between the user interface layer and the execution environment. In conventional bioinformatics applications, graphical interfaces and computational backends are often tightly coupled, rendering analytical workflows vulnerable to changes in operating systems, software libraries, or graphical frameworks. Such fragility poses a particular challenge for genome-based taxonomy, where analyses must remain reproducible across time, users, and computing environments.

To address this limitation, TaxaScope adopts a layered execution model in which the frontend is responsible for parameter configuration, workflow coordination, and progress monitoring, while all genome-based taxonomic analyses are executed within isolated, immutable container environments. As illustrated in Figure 1, the system is organized into four logical layers: (i) a presentation layer providing a graphical user interface for workflow configuration and result inspection, (ii) coordination layer responsible for rule-based task scheduling and controlled execution, (iii) an analysis core composed of containerized bioinformatics tools, and (iv) a host infrastructure layer managing input data and structured output directories. This layered design ensures modularity, portability, and standardized execution environments, which are essential for standardized taxonomic analyses.

### 2.3 User interface and workflow configuration

**Figure 2:**
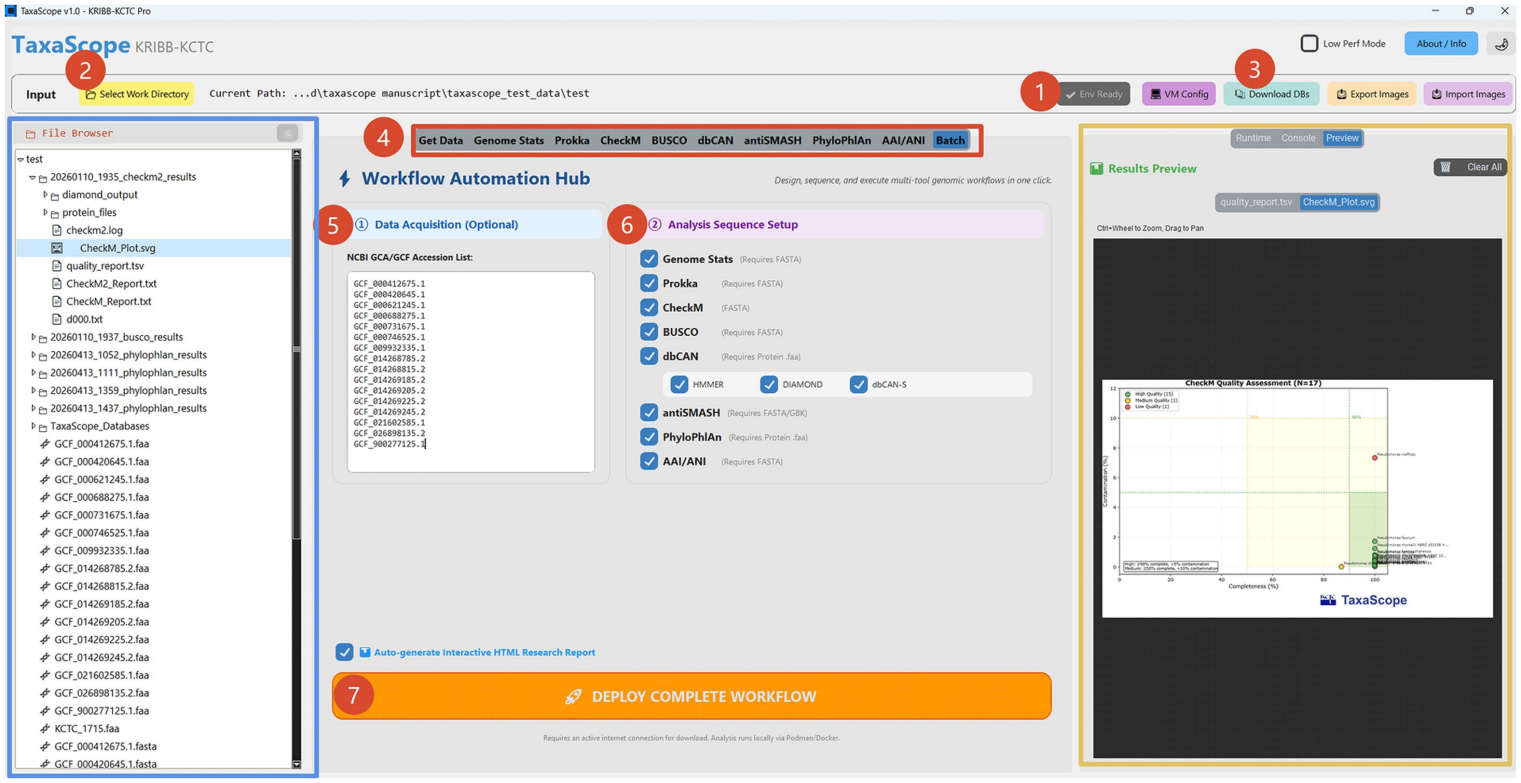
ical user interface (GUI) of TaxaScope and workflow configuration. The TaxaScope graphical user interface provides an integrated environment for configuring and executing genome-based taxonomic workflows. The interface is organized into functional panels guiding users from environment initialization to workflow execution and result inspection. Numbered elements indicate key steps in the workflow: (1) Environment setup. Initialization of the required execution environment, including WSL2 and the container engine (Podman/Docker), performed only once during installation and not required for subsequent analyses. (2) Working directory selection. Users specify a project directory for input data, intermediate files, and outputs. (3) Database download. Required reference databases are downloaded to the working directory, need to be installed only once, and can be reused by relocating them to other working directories. (4) Parameter configuration. Individual analysis modules (e.g., Prokka, CheckM, BUSCO) allow parameter adjustment prior to execution, defining tool-specific settings. (5) Data acquisition (optional). Genome assemblies can be retrieved from NCBI using accession numbers (e.g., GCF/GCA), or alternatively provided as local sequence files (e.g., genome assemblies or protein sequences in FASTA/FA format) placed within the working directory. (6) Workflow selection (batch mode). Users select and combine analysis modules into a sequential workflow. (7) Workflow execution. The pipeline is initiated through a single command. The left panel (blue box) displays the file browser, showing all input files, intermediate results, and outputs generated within the working directory. The central panel presents workflow configuration and module selection. The right panel (yellow box) contains three tabs: (i) Runtime, showing task status and progress; (ii) Console, providing command-line logs for transparency and troubleshooting; and (iii) Preview, enabling visualization of selected results from the file browser, including figures and report outputs.

To facilitate accessibility for users without command-line expertise, particularly experimental microbiologists engaged in bacterial taxonomy, TaxaScope provides a unified graphical user interface (GUI) that serves as the primary entry point for workflow configuration and execution (Figure 2). The interface follows a file-system–centric paradigm, in which users select a working directory containing genome assemblies and associated files, which are automatically detected and organized by the application.

Analysis modules are exposed as modular, task-oriented components that correspond to key steps in genome-based taxonomy. Users may configure individual analyses or combine multiple modules, such as genome quality assessment, annotation, phylogenomic inference, and genome relatedness estimation, into sequential or batch workflows applied to the same dataset. Tool availability and input requirements (e.g., FASTA or protein files) are validated automatically to prevent invalid configurations and ensure methodological consistency.

### 2.4 Containerized execution environment

All computational analyses in TaxaScope are executed within Open Container Initiative (OCI)–compliant containers, with Podman serving as the primary container engine and Docker supported as a fully compatible alternative. Each integrated bioinformatics tool is encapsulated together with its complete runtime environment and reference databases as a version-locked container image. The specific release tags, image digests (SHA256), and build timestamps for all integrated tools are provided in Supplementary Table S1. This approach eliminates the need for users to manually install and maintain complex software dependencies, such as Perl, BioPerl, multiple Python runtimes, Java, HMMER, Prodigal, or BLAST+, on native Windows systems.

When an analysis is initiated, TaxaScope dynamically generates and executes a container launch command corresponding to the selected module. User-specified input and output directories on the host system are mounted into the container as shared volumes, enabling seamless data exchange without manual file transfer. Upon completion, the container is automatically terminated, leaving no residual dependencies or temporary files on the host system. By preserving exact tool versions and runtime configurations, this container-native design supports standardized execution across taxonomic analyses performed at different times or on different machines.

To enable efficient execution of Linux-native bioinformatics tools on Windows systems, TaxaScope leverages the Windows Subsystem for Linux 2 (WSL2), which operates transparently in the background and does not require users to interact directly with Linux-specific environments or command-line configurations. For Windows users, WSL2 is required as part of the container runtime environment and is not bundled within the TaxaScope application; instead, the system provides a guided setup workflow to assist with installation and configuration. This is a one-time setup step and is not required for subsequent analyses.

### 2.5 Workflow orchestration and batch processing

TaxaScope supports both single-genome and batch-based analyses, reflecting common scenarios in genome-based taxonomy where multiple isolates or closely related strains are analyzed in parallel. Users may submit directories containing multiple genome assemblies, which are automatically queued and processed using predefined, tool-specific execution rules.

Task scheduling and concurrency control are handled by the orchestration layer, which applies rule-based execution strategies informed by the computational characteristics of each integrated tool. Computationally intensive or multi-threaded tools are assigned dedicated container instances whereas tools with limited parallelization are executed concurrently across multiple genomes using independent container instances. This rule-based scheduling approach improves overall throughput while maintaining stable execution behavior, without requiring manual tuning by the user.

### 2.6 Integration of core genome analysis modules

TaxaScope integrates a curated set of open-source tools that are central to genome-based bacterial taxonomy. Genome quality assessment is performed using CheckM2 and BUSCO, providing complementary estimates of completeness and contamination. Genome annotation is carried out using Prokka within a containerized environment to ensure consistent behavior across systems. Phylogenomic inference and genome relatedness are assessed using PhyloPhlAn, pyANI, and EzAAI, enabling robust taxonomic placement across both closely related and more divergent genomes. Functional potential is further characterized using antiSMASH for biosynthetic gene cluster detection and dbCAN for carbohydrate-active enzyme annotation, providing additional genomic context commonly reported in taxonomic studies (Seemann, 2014; Simão et al., 2015; Pritchard et al., 2016; Asnicar et al., 2020; Kim et al., 2021; Blin et al., 2023; Chklovski et al., 2023; Zheng et al., 2023). Reference databases used by TaxaScope are not permanently fixed within the container image. Instead, they are downloaded during workflow setup or use, depending on the tool, and can be manually updated by replacing the corresponding files in the designated database directory when newer releases become available.

All tool outputs are automatically collected, parsed, and organized into standardized directory structures, facilitating transparent comparison, downstream analysis, and reproducible visualization, with all intermediate results and associated metadata retained within the output directories for user access.

### 2.7 Automated visualization and output generation

To reduce reliance on manual post-processing and external visualization software, TaxaScope includes an automated visualization engine that operates directly on analysis outputs. Annotation results and sequence-derived metrics are transformed into high-quality graphical summaries suitable for downstream interpretation and figure preparation, including circular genome maps and comparative plots commonly required in taxonomic manuscripts. Visual outputs are exported in scalable vector or high-resolution formats suitable for inclusion in manuscripts and presentations.

### 2.8 Data acquisition via NCBI datasets

To facilitate large-scale comparative genomics, TaxaScope integrates an automated NCBI Data Fetcher module built upon the NCBI Datasets command-line interface. This module enables high-throughput retrieval of genome assemblies (FNA) and corresponding proteomes (FAA) directly using assembly accessions (GCF/GCA), eliminating the need for manual data collection and file organization (Sayers et al., 2022). User-provided accession IDs are passed to the underlying CLI, and the downloaded ncbi_dataset directory is automatically parsed and reorganized for downstream analyses.

### 2.9 Phylogenetic visualization

Phylogenetic trees and integrated genomic datasets were visualized using the TaxaScope visualization engine, which leverages Biopython (Bio.Phylo) for tree topology parsing and Matplotlib for high-quality rendering. Multi-omics metadata (e.g., genome size, GC content, and CAZyme profiles) were aligned to leaf nodes using a dual-axis layout implemented via Pandas and axes_grid1. High-resolution vector outputs (PDF/SVG) were generated to ensure aesthetic precision and biological interpretability (Supplementary Table S2).

## 3 Results

### 3.1 Application example: genome-based taxonomic analysis of *Pseudomonas putida*

To demonstrate the applicability of TaxaScope in a genome-based bacterial taxonomy context, we performed a comprehensive re-analysis of the genome of *Pseudomonas putida*. *P. putida* is a well-characterized Gram-negative bacterium within the phylum *Proteobacteria* and represents an environmentally ubiquitous species with extensive genomic resources, well-defined species boundaries, and multiple curated reference genomes available in public databases (Gomila et al., 2015; Belda et al., 2016). These characteristics make *P. putida* a suitable reference case for demonstrating the integrated workflow, execution consistency, and practical utility of TaxaScope in genome-based bacterial taxonomy.

The genome of *P. putida* is relatively large and information-rich, reflecting its pronounced metabolic versatility and ecological adaptability. Genomes of *P. putida* encode diverse regulatory and metabolic pathways that enable the utilization of a wide range of carbon sources, including aromatic and xenobiotic compounds commonly encountered in soil and rhizosphere environments (Belda et al., 2016; Martínez-García and de Lorenzo, 2019). Owing to its non-pathogenic nature, genetic tractability, and the availability of numerous high-quality reference assemblies and type strains, *P. putida* has been widely adopted as a model organism in environmental microbiology, biotechnology, and genome-based systematics (Nikel and de Lorenzo, 2018). These features make it an appropriate and conservative choice for evaluating whether TaxaScope can reliably integrate genome quality assessment, annotation, phylogenomic placement, and comparative genomic analyses within a unified, consistent taxonomy-oriented workflow.

### 3.2 Dataset acquisition and genome overview

Genome assemblies of *Pseudomonas putida* KCTC 1751ᵀ and related reference strains were retrieved using the NCBI Data Fetcher module of TaxaScope. Genome sequences were batch-downloaded via assembly accession identifiers using the NCBI Datasets command-line interface (CLI, version 2), the officially recommended tool for programmatic access to NCBI genome resources (Sayers et al., 2022). All assemblies were automatically organized into a standardized project directory, ensuring consistent data provenance.

To summarize the genome dataset, TaxaScope generated a genome statistics overview for all imported assemblies, including total genome length, contig number, GC content, and N50 values. Organism names and strain designations were automatically linked to each assembly based on associated metadata. The resulting genome overview table (Table 1) provides a standardized snapshot of the dataset used for subsequent genome quality assessment and comparative taxonomic analyses.

**Table 1:** Genome assemblies and basic genomic features of strains analyzed in this study.

Organism name	Strain/isolate	Accession	Total length (bp)	Contigs	GC content (%)	N50
*P. putida*	NBRC 14164ᵀ	GCF_000412675.1	6,156,701	1	62.33	6,156,701
*D.caeni*	DSM 24390ᵀ	GCF_000421765.1	3,022,325	24	48.26	312,453
*P. taiwanensis*	DSM 21245ᵀ	GCF_000425785.1	5,415,134	67	61.84	174,759
*P. monteilii*	NBRC 103158ᵀ	GCF_000621245.1	6,308,713	85	61.49	204,302
*P. plecoglossicida*	NBRC 103162 ᵀ	GCF_000688275.1	5,347,571	58	62.98	172,824
*P. capeferrum*	WCS358ᵀ	GCF_000731675.1	5,940,443	8	62.63	1,147,469
*P. alkylphenolica*	KL28ᵀ	GCF_000746525.1	5,764,622	1	60.63	5,764,622
*P. huaxiensis*	WCHPs060044ᵀ	GCF_003231275.1	7,264,993	241	62.26	141,882
*P. asiatica*	RYU5ᵀ	GCF_009932335.1	5,969,036	3	62.55	4,196,743
*P. shirazensis*	SWRI56ᵀ	GCF_014268785.2	4,753,349	19	61.85	2,451,732
*P. farsensis*	SWRI107ᵀ	GCF_014268805.2	5,249,961	6	62.58	1,525,682
*P. urmiensis*	SWRI10ᵀ	GCF_014268815.2	5,786,335	9	61.81	5,577,211
*P. promysalinigenes*	RW10S1ᵀ	GCF_014269025.2	5,046,521	1	60.62	5,046,521
*P. vlassakiae*	RW4S2ᵀ	GCF_014269035.2	5,632,542	80	62.97	224,431
*P. oryzicola*	RD9SR1ᵀ	GCF_014269185.2	5,250,451	24	62.91	3,915,385
*P. kermanshahensis*	SWRI100ᵀ	GCF_014269205.2	6,146,916	24	62.22	5,463,999
*P. anuradhapurensis*	RD8MR3ᵀ	GCF_014269225.2	5,393,636	1	63.43	5,393,636
*P. kurunegalensis*	RW1P2ᵀ	GCF_014269245.2	5,364,230	61	62.09	279,406
*P. faucium*	BML-PP048ᵀ	GCF_021602585.1	5,894,173	89	64.19	219,428
*P. fortuita*	GMI12077ᵀ	GCF_026898135.2	6,311,744	1	62.02	6,311,744
*P. fontis*	ID656ᵀ	GCF_028656975.1	6,074,957	157	61.93	81,842
*P. asplenii*	ATCC 23835ᵀ	GCF_900105475.1	6,529,636	1	61.22	6,529,636
*P. inefficax*	JV551A3ᵀ	GCF_900277125.1	6,240,036	248	62.85	134,841
*P. putida*	KCTC_1751ᵀ	GCF_024508115.1	6,179,370	1	62.33	6,179,370

### 3.3 Quality control assessment

In genome-based bacterial taxonomy, rigorous genome quality assessment constitutes a critical gatekeeping step, as incomplete or contaminated assemblies can compromise species delineation, phylogenomic inference, and comparative genomic analyses. We therefore applied the Quality Control module of TaxaScope to evaluate the integrity and suitability of all genomes prior to downstream taxonomic analyses. The analyzed dataset included genomes with heterogeneous assembly qualities, including fragmented draft genomes with dozens to hundreds of contigs and assemblies with elevated contamination or reduced completeness, thereby reflecting common datasets encountered in genome-based bacterial taxonomy.

**Figure 3:**
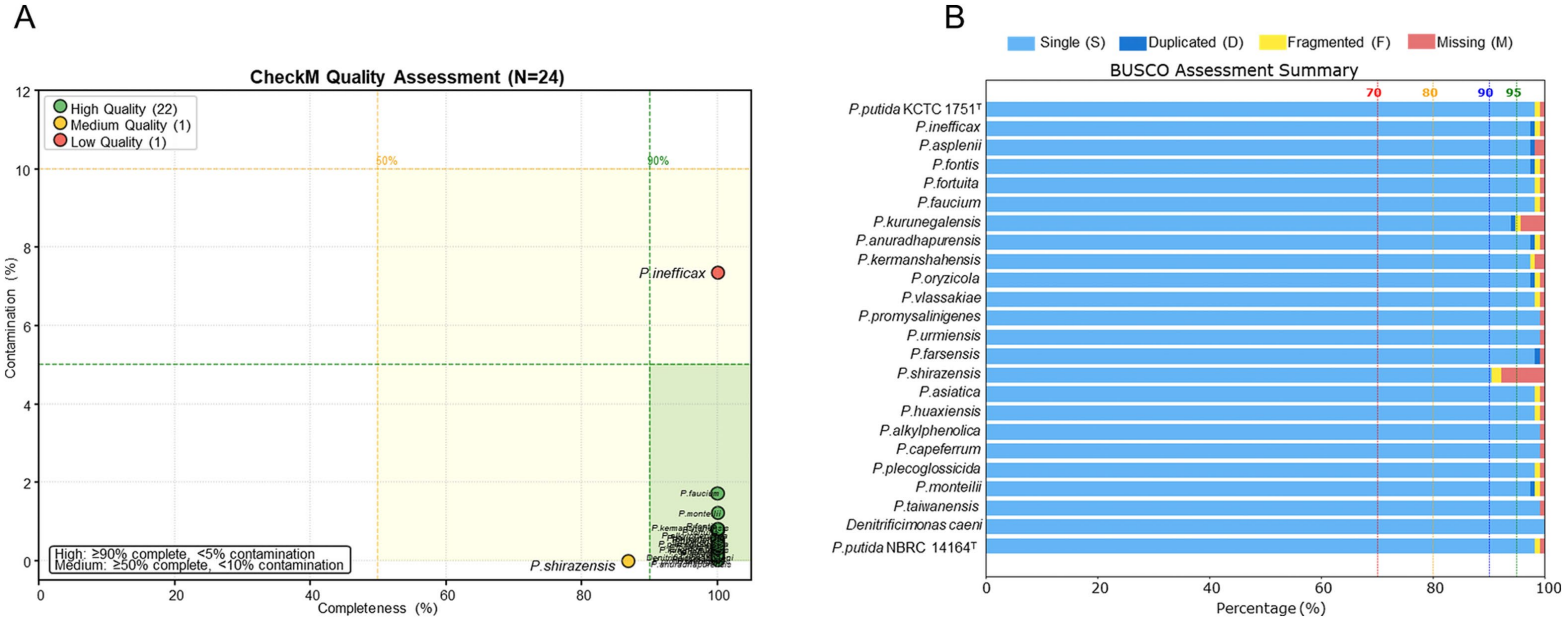
Genome quality assessment of the analyzed genomes using CheckM2 and BUSCO. **(A)** CheckM2-based evaluation of genome completeness and contamination for the 24 analyzed genomes. Each point represents one genome, plotted according to estimated completeness (%) and contamination (%). Dashed lines indicate commonly used quality thresholds, with high-quality genomes defined as having >90% completeness and <5% contamination. **(B)** BUSCO assessment summary based on the bacterial lineage dataset, showing the proportions of single-copy (S), duplicated (D), fragmented (F), and missing (M) BUSCO genes for each genome. Vertical dashed reference lines indicate completeness thresholds at 70, 80, 90, and 95%.

Genome quality assessment was performed using CheckM2 with the standard bacterial lineage-specific marker set. Most genomes were classified as high quality, exhibiting high completeness (>90%) and low contamination (<5%; Figure 3A, Supplementary Table S3). Notably, one genome (*P. inefficax*) showed an elevated contamination level, while P. shirazensis displayed reduced completeness, clearly separating these assemblies from the main high-quality genome cluster. In contrast, *P. putida* KCTC 1751^T^, which serves as the focal genome for downstream taxonomic demonstration, exhibited 100.0% completeness and only 0.4% contamination, indicating a near-complete and highly reliable assembly. Similarly, the reference strain *P. putida* NBRC 14164ᵀ also showed 100.0% completeness with a contamination level of 0.41%, providing a robust benchmark for species-level comparative analyses.

To independently corroborate genome completeness, BUSCO v5 analysis using the bacteria_odb12 lineage dataset was conducted for the same genome set. Consistent with the CheckM2 results, *P. shirazensis*, which exhibited lower completeness in CheckM2, also showed an increased proportion of missing and fragmented BUSCOs (Figure 3B, Supplementary Table S3), whereas *P. putida* KCTC 1751^T^ and *P. putida* NBRC 14164ᵀ both achieved 100% complete BUSCO scores, confirming the high integrity of these assemblies. The concordant results obtained from CheckM2 and BUSCO indicate that TaxaScope provides a standardized environment for batch-based genome quality assessment while including both high-confidence reference genomes and lower-quality assemblies that may affect downstream phylogenomic and taxonomic analyses.

### 3.4 Phylogenomic placement and genomic identity

**Figure 4:**
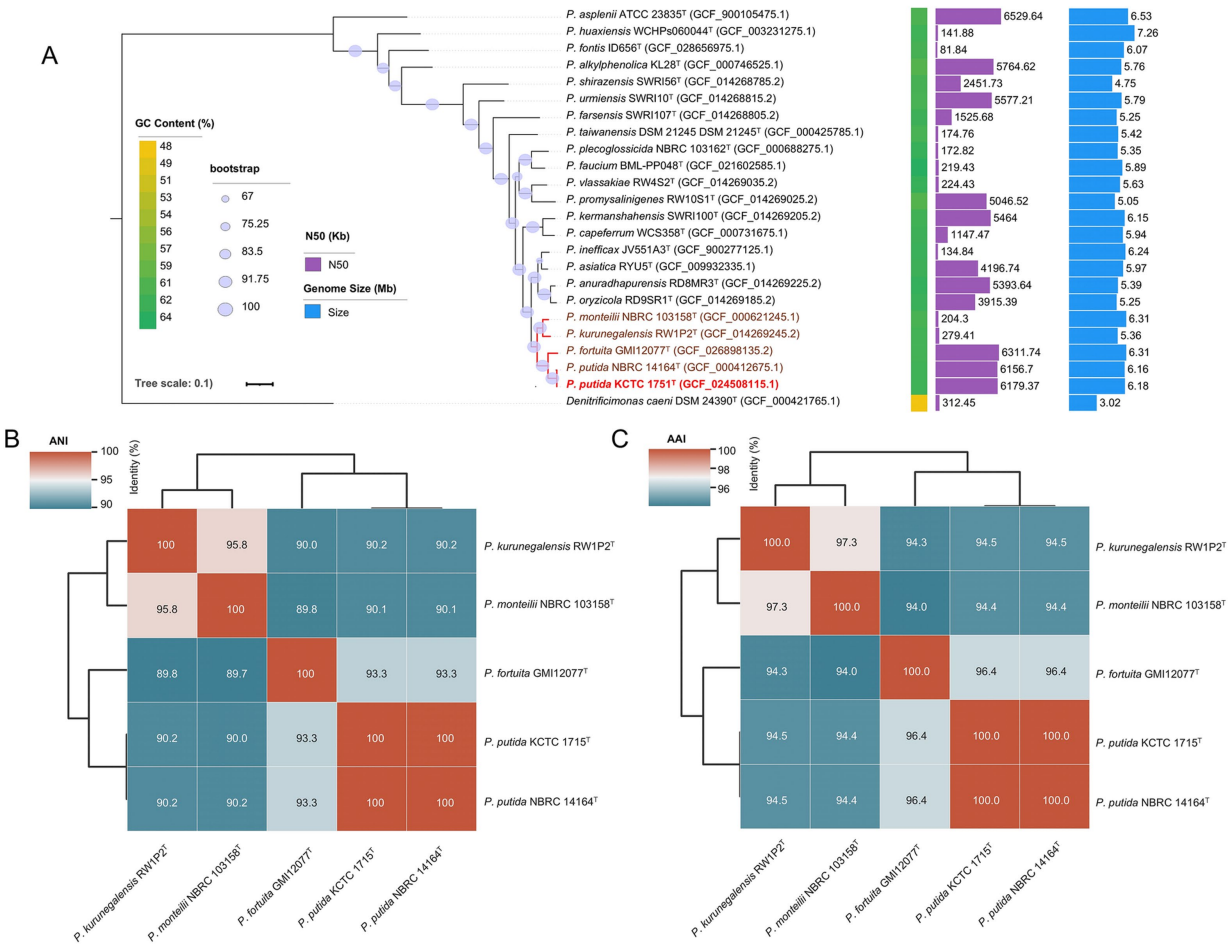
Phylogenomic placement and genome similarity analysis of the analyzed genomes. **(A)** Whole-genome phylogenetic tree inferred using PhyloPhlAn based on conserved marker genes. The tree is annotated with genome statistics, including GC content (%), number of contigs, total genome length (Mb), and N50 (kb). **(B)** Average nucleotide identity (ANI) matrix and corresponding clustering, showing nucleotide-level genome similarity among the analyzed genomes. **(C)** Average amino acid identity (AAI) matrix and hierarchical clustering among the analyzed genomes, illustrating protein-level similarity patterns across taxa. Color scales indicate pairwise identity values. Together, these analyses provide a multi-level overview of phylogenetic relationships and genomic relatedness within the dataset.

In genome-based bacterial taxonomy, phylogenomic placement provides the primary evolutionary framework upon which species boundaries are evaluated using quantitative genome similarity metrics. To establish this framework, a phylogenomic tree was inferred using PhyloPhlAn based on conserved marker genes. Following tree inference, TaxaScope generated an interactive preview and automatically exported iTOL-compatible annotation files, enabling the integration of genome-level metadata into phylogenetic visualization. With minimal manual adjustment in iTOL, a high-quality phylogenomic tree suitable for final presentation was obtained, annotated with GC content, genome length, contig number, and N50 values (Figure 4A).

Phylogenomic analysis showed that *P. putida* KCTC 1751ᵀ clustered tightly with *P. putida* NBRC 14164ᵀ, forming a well-supported monophyletic group with strong bootstrap support. The bootstrap support values shown in Figure 4A indicate statistical support for this clustering. This close relationship is consistent with their assignment to the same species and supports the taxonomic placement of KCTC 1751ᵀ within the *P. putida* species.

In addition, *P. putida* KCTC 1751ᵀ and *P. putida* NBRC 14164ᵀ were embedded within a larger, coherent clade comprising *P. monteilii* NBRC 103158ᵀ, *P. kurunegalensis* RW1P2ᵀ, and *P. fortuita* GMI12077ᵀ. This higher-level cluster corresponds to the *P. putida* species group and delineates a closely related lineage within the genus *Pseudomonas*. Based on this phylogenomic framework, a subset of 5 closely related genomes (KCTC 1751ᵀ, NBRC 14164ᵀ, NBRC 103158ᵀ, RW1P2ᵀ, and GMI12077ᵀ) was selected, and subsequent comparative analyses were focused on this clade to examine genome similarity metrics and functional feature variation among closely related taxa.

To quantitatively evaluate species-level boundaries, average nucleotide identity (ANI) analysis was performed for genomes within the *P. putida* species group. As expected, *P. putida* KCTC 1751ᵀ exhibited ANI values of 100% with *P. putida* NBRC 14164ᵀ, confirming their conspecific relationship. In contrast, ANI values between *P. putida* KCTC 1751ᵀ and other closely related species were all below the widely accepted species threshold of approximately 95–96%, supporting their separation at the species level (Richter and Rosselló-Móra, 2009; Figure 4B).

To further assess genomic relatedness beyond nucleotide-level similarity, average amino acid identity (AAI) was calculated for the same genome set. While AAI values between *P. putida* strains reached 100%, interspecies comparisons within the *P. putida* species group yielded AAI values exceeding 94%, reflecting close evolutionary relationships at the species-group level rather than violation of species boundaries (Barco et al., 2020; Figure 4C). Together, the concordant phylogenomic placement and ANI/AAI patterns demonstrate a coherent and internally consistent framework for genome-based taxonomic inference.

### 3.5 Genome annotation and automated visualization

Following quality control, the genomes were annotated using the Prokka module integrated within TaxaScope to generate a standardized gene annotation framework suitable for genome-based taxonomic comparison.

**Figure 5:**
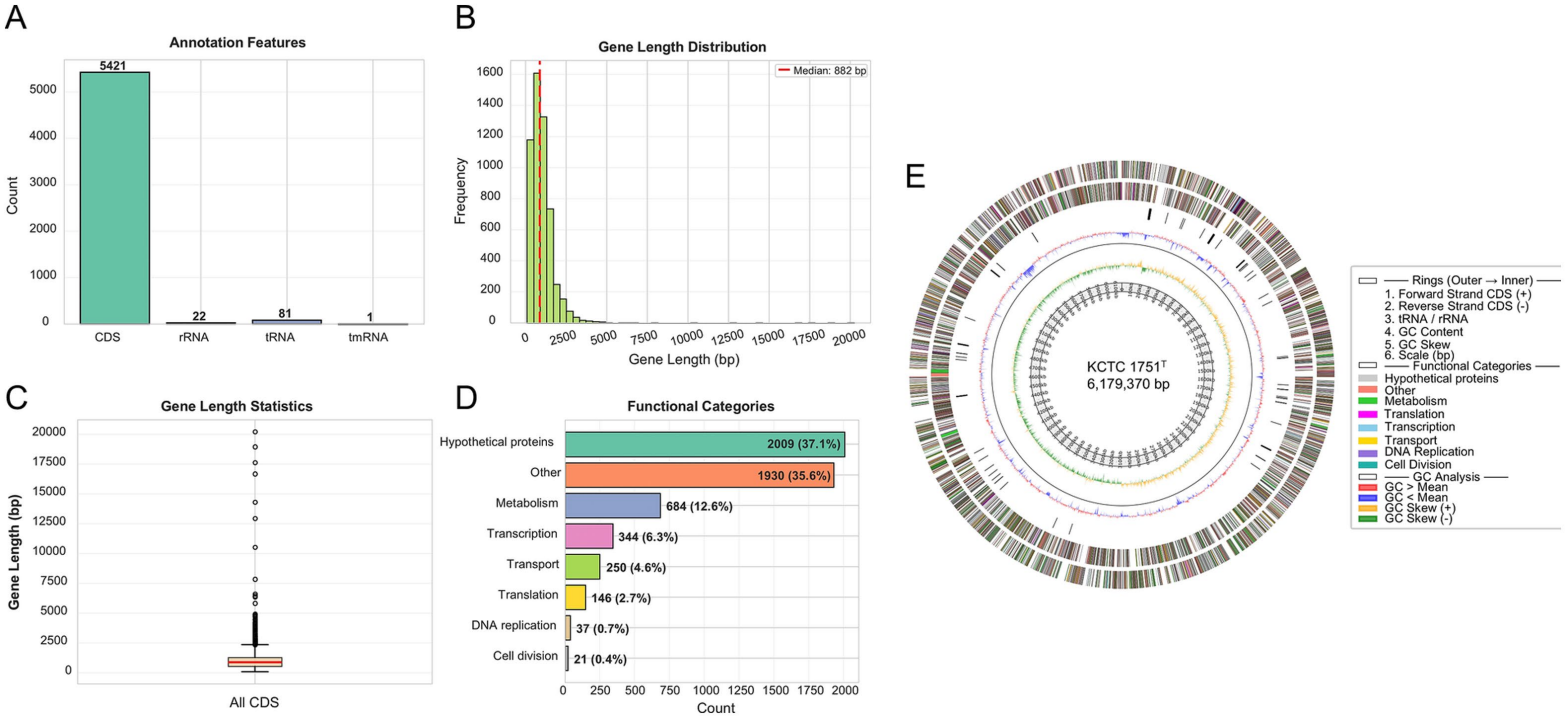
Genome annotation summary and automated visualization for *Pseudomonas putida* KCTC 1751^T^. **(A)** Annotation feature counts predicted by the containerized Prokka workflow, including protein-coding sequences (CDSs), rRNAs, tRNAs, and tmRNA. **(B)** Distribution of CDS lengths showing a right-skewed profile typical of bacterial genomes; the dashed red line indicates the median CDS length (882 bp). **(C)** Boxplot representation of CDS length statistics, illustrating the compact size distribution of most genes and the presence of a limited number of longer CDSs. **(D)** Functional classification of predicted proteins based on annotation categories, with counts and proportions shown for major functional groups. **(E)** Circular genome overview plot automatically generated by TaxaScope for the reference assembly (KCTC 1751^T^; 6,179,370 bp). From outer to inner rings, the plot displays protein-coding genes on the forward and reverse strands, tRNA and rRNA locations, GC content, GC skew, and genomic scale. Genes are color-coded by functional category, providing an integrated visualization of genome organization and compositional features.

Genome annotation was performed using the containerized version of Prokka. For *P. putida* KCTC 1751ᵀ, Prokka predicted 5,421 protein-coding sequences (CDSs), together with 81 tRNAs, 22 rRNAs, and one tmRNA (Figure 5A). These values are highly consistent with those obtained for the reference strain *P. putida* NBRC 14164ᵀ, for which nearly identical gene counts and RNA features were observed (Supplementary Figure S1). The close agreement between these two independently sequenced strains indicates that the Prokka workflow implemented in TaxaScope yields stable and comparable annotations suitable for genome-based taxonomic analysis. Analysis of CDS length distributions revealed a right-skewed profile characteristic of bacterial genomes, with a median CDS length of 882 bp (Figure 5B). A comparable distribution pattern was observed in *P. putida* NBRC 14164ᵀ (Supplementary Figure S2B). Boxplot visualization further showed that most genes clustered within a compact size range, while a limited number of longer CDSs formed the upper tail of the distribution (Figure 5C). Comparable CDS length distributions were observed for *P. putida* NBRC 14164ᵀ, further supporting annotation consistency across strains (Supplementary Figure S1).

Functional classification of the predicted proteins indicated that 37.1% were annotated as hypothetical proteins, while an additional 35.6% were assigned to other broad and functionally heterogeneous categories that could not be readily grouped into major biological processes. This functional composition closely mirrors that observed in *P. putida* NBRC 14164ᵀ (Supplementary Figure S1) and reflects both genuine genetic novelty and the intrinsic limitations of current annotation frameworks commonly reported for *P. putida*. Among the more specifically annotated functional classes, genes related to metabolism (12.6%), transcription (6.3%), and transport (4.6%) were prominently represented, consistent with the well-recognized metabolic versatility of this species (Figure 5D).

Upon completion of annotation, TaxaScope automatically generated a circular genome overview plot as the default visualization output (Figure 5E and Supplementary Figure S1). This plot summarizes key genomic features of the reference assembly (KCTC 1751^T^; 6,179,370 bp), including the distribution of protein-coding genes on the forward and reverse strands, the locations of tRNA and rRNA genes, and genome-wide profiles of GC content and GC skew. By combining annotation and compositional features into a single visualization, this automated output provides a concise, high-quality representation of genome organization suitable for downstream interpretation and figure preparation, commonly required in taxonomic manuscripts, without the need for manual post-processing.

### 3.6 Secondary metabolite mining

To provide supportive genomic context commonly reported in taxonomic studies, the antiSMASH v8 module integrated in TaxaScope was applied to identify biosynthetic gene clusters (BGCs) in *Pseudomonas putida*. The predicted BGCs encompassed multiple major classes, including NRPS and NRPS-related clusters (e.g., NRPS-metallophore), RiPP-like clusters, NAGGN-associated clusters, non-iron siderophore pathways, as well as additional functional types such as arylpolyene, acyl–amino acid, ranthipeptide, redox-cofactor, hydrogen cyanide, and terpene precursor clusters. This diversity of BGC classes is consistent with the well-documented secondary metabolic potential of *P. putida* and closely related taxa, indicating that the TaxaScope workflow can recover expected biosynthetic profiles for well-characterized strains.

**Figure 6:**
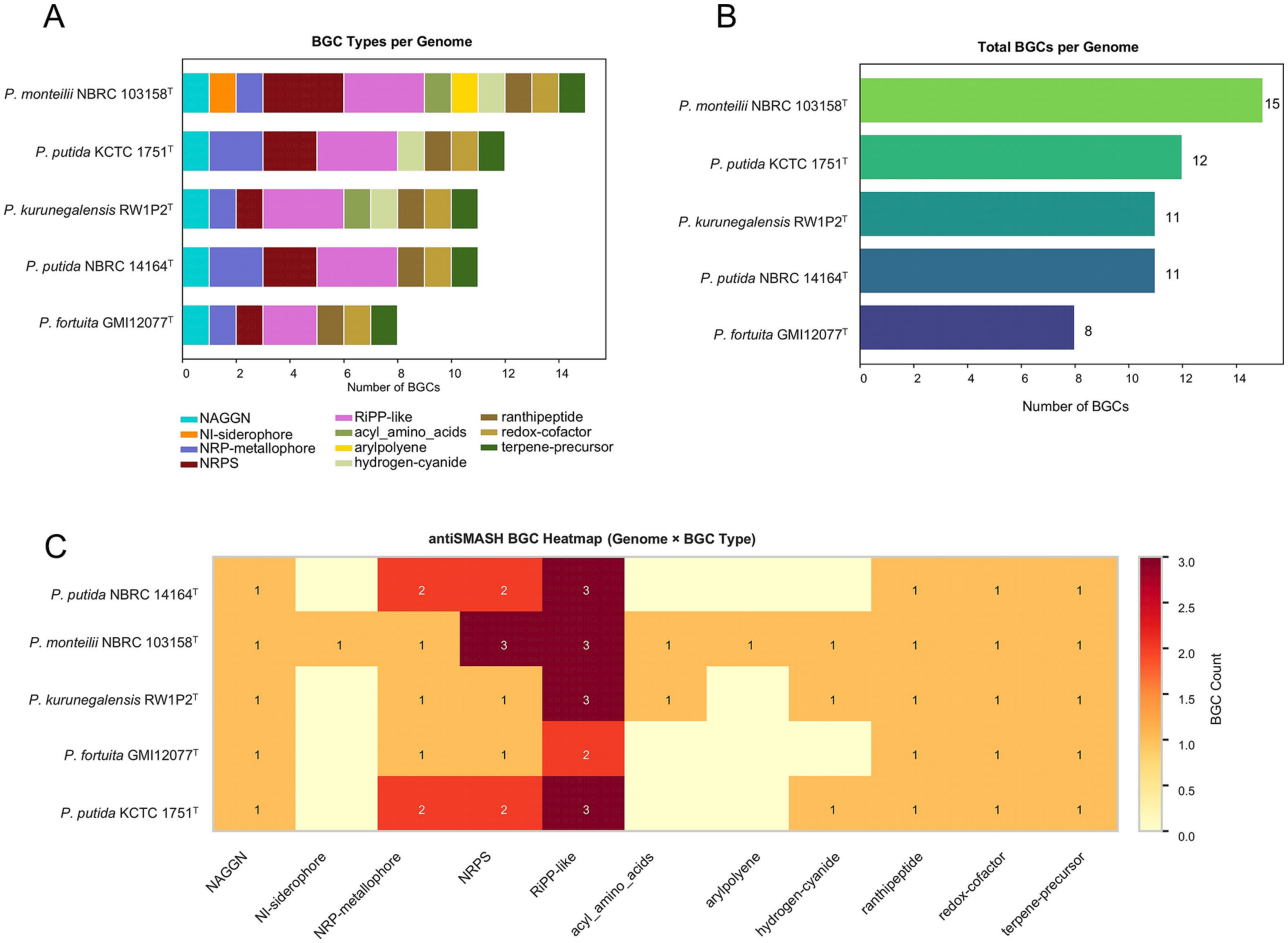
Comparative analysis of biosynthetic gene clusters (BGCs) identified by antiSMASH across the analyzed genomes. **(A)** Distribution of predicted BGC types per genome, showing the composition and relative abundance of different biosynthetic classes identified by antiSMASH v8 under relaxed detection settings. **(B)** Total number of BGCs detected in each genome. *Pseudomonas putida* KCTC 1751^T^ harbors 12 predicted BGCs, placing it among the genomes with a relatively high biosynthetic potential in this dataset. **(C)** Heatmap representation of BGC counts across genomes and biosynthetic classes, illustrating the diversity and distribution patterns of secondary metabolite gene clusters. Color intensity reflects the number of BGCs detected for each class–genome combination.

Notably, comparative analysis revealed a high degree of concordance between *P. putida* KCTC 1751ᵀ and the reference strain *P. putida* NBRC 14164ᵀ with respect to both the total number and class composition of predicted BGCs (Figures 6A–C). Differences between the two strains were limited to minor quantitative variation in specific BGC subclasses, while all major biosynthetic categories were shared. Such variation is consistent with strain-level diversity commonly observed among independently sequenced *P. putida* isolates and is consistent with previously reported strain-level diversity.

For each analyzed genome, TaxaScope automatically generated both static summary plots and an interactive antiSMASH HTML report using antiSMASH v8 in relaxed detection mode. These outputs enable direct inspection of predicted BGC boundaries, domain architectures, and annotations, while preserving fully local execution without reliance on external web servers, data upload, or manual file handling. Comparative summaries of BGC counts and classes across genomes were compiled automatically, facilitating consistent reporting within genome-based studies. In TaxaScope, multiple antiSMASH parameter settings are supported, and the relaxed mode was selected here for demonstration purposes, although it may increase sensitivity and potentially introduce false-positive predictions; therefore, parameter selection should be adapted to the specific dataset.

### 3.7 Carbohydrate-active enzyme (CAZyme) analysis

**Figure 7:**
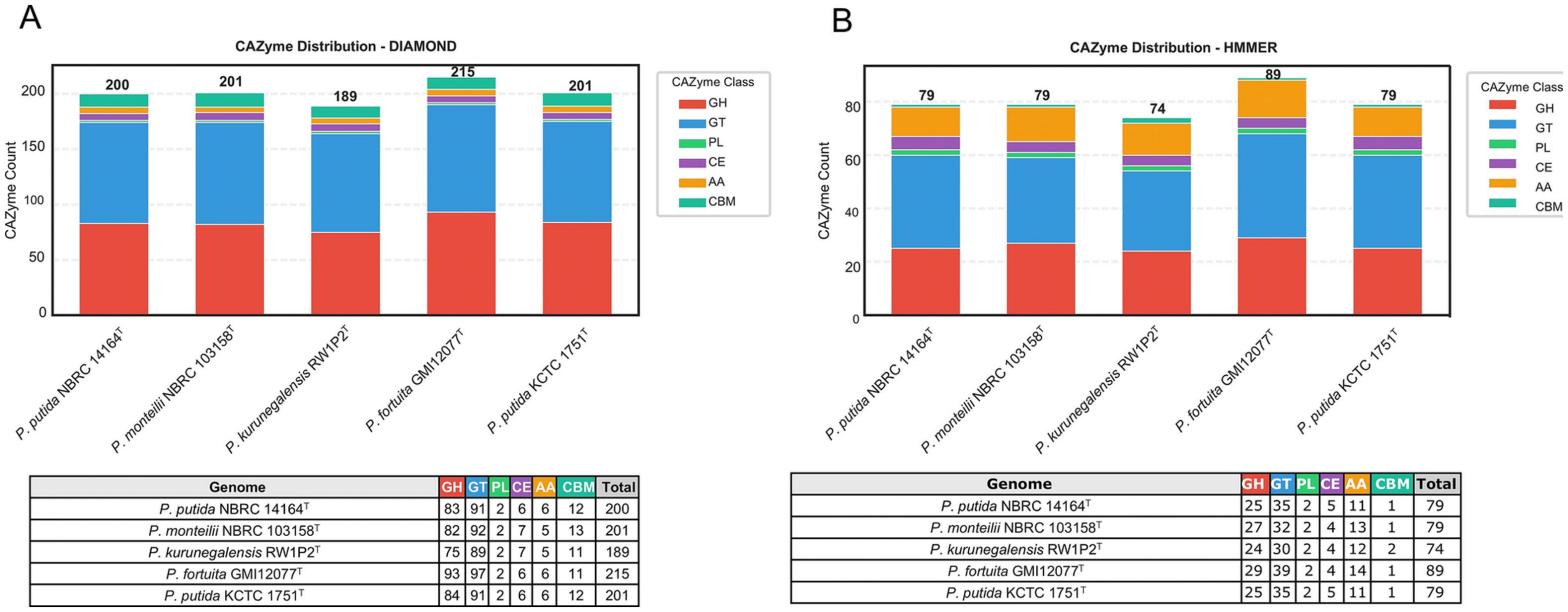
Carbohydrate-active enzyme (CAZyme) profiles inferred using the dbCAN pipeline. **(A)** Distribution of CAZyme families identified using the DIAMOND-based annotation strategy across the analyzed genomes. Stacked bars represent counts of major CAZyme classes, including glycoside hydrolases (GH), glycosyltransferases (GT), polysaccharide lyases (PL), carbohydrate esterases (CE), auxiliary activities (AA), and carbohydrate-binding modules (CBM). **(B)** CAZyme distribution inferred using the HMMER-based annotation approach, applying hidden Markov model profiles for more stringent detection. For *Pseudomonas putida* KCTC 1751^T^, DIAMOND-based annotation identified over 201 putative CAZymes, whereas the HMMER-based method yielded a more conservative set of approximately 79 CAZymes, reflecting methodological differences in sensitivity and specificity between the two approaches.

To provide supplementary functional context for comparative genomic interpretation in a taxonomic framework, the dbCAN module was applied to annotate carbohydrate-active enzymes (CAZymes). For *P. putida* KCTC 1751ᵀ, dbCAN identified a diverse CAZyme repertoire, with glycoside hydrolases (GH) and glycosyltransferases (GT) representing the most abundant classes in both DIAMOND- and HMMER-based predictions (Figure 7). Additional CAZyme classes, including carbohydrate esterases (CE), polysaccharide lyases (PL), auxiliary activities (AA), and carbohydrate-binding modules (CBM), were detected at lower frequencies.

Consistent trends were observed between the two annotation strategies, with DIAMOND yielding higher total CAZyme counts (Figure 7A) and HMMER providing a more conservative but concordant class distribution (Figure 7B), reflecting the complementary nature of sequence similarity– and profile-based approaches. Importantly, the overall CAZyme class composition and relative abundance patterns observed for *P. putida* KCTC 1751ᵀ were highly consistent with those of the reference strain *P. putida* NBRC 14164ᵀ, with only minor quantitative variation across specific CAZyme families. This close agreement supports the consistency and reliability of the dbCAN workflow implemented in TaxaScope for comparative, taxonomy-oriented functional profiling.

TaxaScope automatically integrated and summarized CAZyme annotation outputs at the genome level, enabling standardized and reproducible comparison of CAZyme class composition across genomes without requiring manual parsing or post-processing.

## 4 Discussion

### 4.1 The imperative of local, reproducible computing

The case study based on *Pseudomonas putida* KCTC 1751ᵀ highlights a central methodological advantage of TaxaScope in the context of genome-based bacterial taxonomy: enabling comprehensive and standardized taxonomic analyses to be conducted locally with minimal computational overhead for end users. Importantly, the genomic characteristics inferred for *P. putida* KCTC 1751ᵀ in this study are consistent with previously reported genome-based descriptions of the species. In particular, both genome size and GC content fall within the ranges reported for well-characterized *P. putida* reference strains, including KT2440 and the type strain *P. putida* NBRC 14164ᵀ, whose genomes are approximately 6.1–6.3 Mb in size with GC contents of ~61–62% according to established genome analyses (Nelson et al., 2002; Belda et al., 2016). This concordance suggesting that the integrated tools execute correctly within the TaxaScope environment.

In conventional practice, performing and reproducing genome-based taxonomic analyses across studies typically requires the establishment of a dedicated Linux environment, coordination of multiple bioinformatics tools and reference databases, and the development of custom scripts for result parsing and visualization. Such complexity is particularly problematic for taxonomic studies, where analytical consistency across time, users, and laboratories is essential for species delineation and comparative interpretation, yet workflows are often vulnerable to software incompatibilities, commonly referred to as “dependency hell,” that can compromise execution consistency (Wratten et al., 2021; Boettiger, 2015).

TaxaScope addresses these challenges through a container-based execution. By encapsulating each bioinformatics tool together with its complete runtime environment into isolated, version-controlled containers, TaxaScope establishes a standardized, portable computational environment. Consequently, analyses performed using the same TaxaScope configuration and container versions can be reproduced reliably by different users and across diverse computing environments, supporting consistent and reproducible execution of key analyses such as ANI/AAI-based genome similarity estimation, phylogenomic reconstruction, and genome annotation–based functional characterization in genome-based bacterial taxonomy (Chun et al., 2018).

### 4.2 Performance considerations: local execution and cloud-based platforms

Cloud-based platforms such as Galaxy and KBase provide powerful and widely adopted infrastructures for large-scale and collaborative bioinformatics analyses (Afgan et al., 2018; Arkin et al., 2018). However, as shared computational resources, they may involve usage quotas, job scheduling policies, and queue times (Leipzig, 2017). In the context of genome-based bacterial taxonomy, where analyses are often performed repeatedly on a limited number of genomes for species delineation and comparative evaluation, such factors may reflect different operational characteristics across platforms, and highlight the need for flexible, locally deployable solutions in certain use scenarios.

TaxaScope is designed to execute taxonomic workflows directly on local hardware, enabling analyses to be initiated directly within a Windows desktop environment, typically for workflows involving a moderate number of genomes. In the *P. putida* KCTC 1751ᵀ application example, the complete workflow, from genome import through phylogenomic inference and automated visualization, was completed efficiently on a standard consumer-grade desktop, with containerized execution under WSL2 providing a consistent runtime environment, although it may introduce additional resource overhead compared to native Linux systems, it remains suitable for workflows involving a moderate number of genomes on standard consumer-grade desktop systems. Under these conditions, the resulting phylogenomic placement and genome similarity metrics are consistent with previously established phylogenomic frameworks for the *P. putida* species complex, in which core genome–based analyses provide stable and reproducible taxonomic resolution across closely related strains (Peix et al., 2018).

Importantly, local execution also allows analyses to be performed without transferring genomic data to external servers, a consideration of particular relevance for analyses involving clinical isolates, proprietary strain collections, or datasets subject to institutional, ethical, or regulatory data governance requirements (Schatz et al., 2010; Stephens et al., 2015). Concerns related to data sovereignty, patient privacy, and long-term data control have increasingly influenced the choice of computational platforms in microbial genomics and clinical microbiology (Voelkerding et al., 2009). In this regard, TaxaScope is intended as a complementary local solution that can be used alongside cloud-based platforms, depending on specific analytical needs and use scenarios.

### 4.3 Cost–benefit considerations in genome-based taxonomy

Commercial software suites such as CLC Genomics Workbench and Geneious, which typically require substantial annual licensing fees, TaxaScope is implemented as a cost-free solution for core genome-based taxonomic workflows. Commercial platforms offer polished graphical interfaces and extensive manual editing functionalities, such as primer design, sequence manipulation, and interactive curation, which are commonly used in exploratory or teaching-oriented applications (Kearse et al., 2012).

TaxaScope is designed to support automated, standardized, and reproducible analyses based on community-validated open-source tools. Its primary focus lies in workflow transparency, version control, and batch-oriented execution rather than fine-grained manual sequence manipulation. This design is consistent with methodological priorities of genome-based bacterial taxonomy, where consistent analytical pipelines are essential for species delineation and comparative interpretation across studies (Parks et al., 2018).

Minor discrepancies observed between annotation statistics or functional feature counts obtained in this study and values reported in earlier *Pseudomonas putida* genome analyses are therefore likely attributable to expected consequences of database updates and software version differences rather than biological inconsistency. Such variation has been explicitly documented in successive re-annotations of *P. putida* reference genomes and is a recognized feature of modern genome-based analyses (Nelson et al., 2002; Belda et al., 2016). TaxaScope preserves version-controlled execution environments, allowing these differences to be transparently documented, reproduced, and interpreted within a taxonomic framework.

### 4.4 Advantages, limitations, and appropriate use cases of TaxaScope

It is important to acknowledge that TaxaScope operates within the computational constraints of the host machine. While the platform substantially lowers the technical barrier to genome-based bacterial taxonomy, it does not eliminate intrinsic hardware limitations associated with local computing environments. Analyses involving highly memory-intensive tools, such as GTDB-Tk, or projects requiring the concurrent processing of hundreds to thousands of genomes remain better suited to high-performance computing (HPC) infrastructures or cloud-based platforms (Chaumeil et al., 2019).

Nevertheless, for the majority of microbiologists engaged in genome-based taxonomy, who typically work with isolate genomes or moderately sized comparative datasets, TaxaScope provides a practical and well-balanced solution. By prioritizing standardized execution, data privacy, and workflow consistency, the platform is particularly well suited for routine taxonomic analyses as well as exploratory taxonomic investigations conducted in local laboratory computing environments. In addition, TaxaScope enables automated batch execution of eight genome analysis modules and generates structured reports in JSON, Markdown, and HTML formats that include key results, software versions, database versions, runtime information, and method summaries for result interpretation and reproducible reporting. Representative reports are available in the TaxaScope GitHub repository. Thus, the main contribution of TaxaScope lies not in improving the computational performance or algorithmic accuracy of the underlying tools, but in integrating fragmented command-line–based taxonomic workflows into a unified, GUI-based, and reproducible local analysis platform for bacterial taxonomists and experimental microbiologists. Compared with cloud-based platforms and conventional command-line workflows, TaxaScope therefore emphasizes local data control, reduced dependency-management burden, GUI-based accessibility, and taxonomy-oriented workflow integration for bacterial taxonomists and experimental researchers (Supplementary Table S4).

Accordingly, the present study evaluates TaxaScope primarily as a software workstation for reproducible execution and reporting, rather than as a benchmark study of genome-based taxonomic methodology.

## 5 Conclusion

TaxaScope represents a practical methodological advance toward making genome-based bacterial taxonomy accessible, reproducible, and standardized in wet-lab research environments. By encapsulating widely adopted, command-line–driven bioinformatics tools within a container-native and user-friendly desktop application, TaxaScope lowers the technical barriers that have historically limited the routine adoption of genome-scale analyses in taxonomic studies. The high-fidelity re-analysis of *P. putida* KCTC 1751^T^ demonstrates that core taxonomic workflows, including genome quality assessment, standardized annotation, phylogenomic placement, and quantitative genome similarity analysis, can be executed locally in a consistent and consistent manner, as demonstrated using a well-characterized reference strain with known genomic properties whose expected outputs are well-documented in the literature. Importantly, TaxaScope also bridges the final gap between analysis and reporting by automatically generating high-quality visualizations suitable for downstream interpretation and figure preparation, reducing the need for custom scripting or external visualization tools. As genome sequencing continues to reshape bacterial systematics, platforms such as TaxaScope will play an important role in supporting consistent, transparent, and reproducible taxonomic practice across laboratories. Future development will focus on extending the platform to additional data modalities and workflows, while maintaining its core design philosophy: enabling genome-based taxonomy through standardized execution, local control, and methodological clarity.

## Data Availability

The genome sequences analyzed in this study are publicly available in the GenBank database. The accession numbers and strain information for all analyzed genomes, including Pseudomonas putida KCTC 1751^T^, are provided in Table 1. TaxaScope is freely available at https://github.com/pyx647/TaxaScope-KRIBB-KCTC. A detailed step-by-step user manual, including an end-to-end workflow tutorial and reproducibility reporting guidelines, is also available in the GitHub repository. All genome assemblies analyzed in this study were retrieved from the NCBI Assembly database using the NCBI Datasets command-line interface integrated into TaxaScope.
