# Supplementary material for: TaxaScope: a container-native, visualization-centric workstation for genome-based bacterial taxonomy
Source: Front Microbiol. 2026 May 29;17:1809734. doi: 10.3389/fmicb.2026.1809734 (PMC13260571; doi:10.3389/fmicb.2026.1809734)
Supplement: Supplementary file 1 [file Data_Sheet_1.pdf]

# Supplementary materials

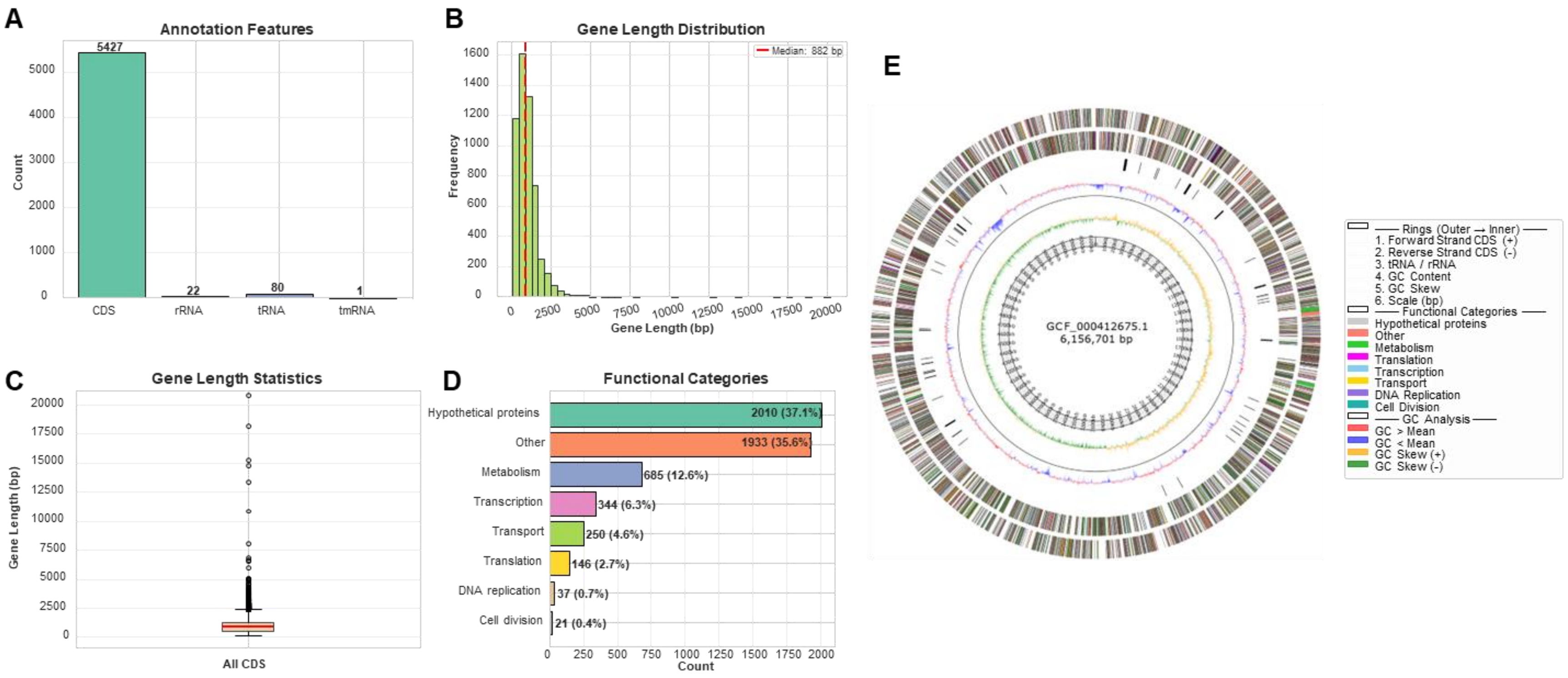


**Figure S1. Genome annotation summary and automated visualization for *Pseudomonas putida* NBRC 14164$^{T}$.** (A) Summary of annotation features generated by the containerized Prokka pipeline, including protein-coding sequences (CDSs), rRNAs, tRNAs, and tmRNA. (B) Length distribution of CDSs, showing a right-skewed pattern typical of bacterial genomes; the dashed red line marks the median CDS length (882 bp). (C) Boxplot of CDS length statistics, highlighting the generally compact gene size distribution along with a small proportion of longer CDSs. (D) Functional categorization of predicted proteins, with counts and relative proportions for major annotation classes. (E) Circular genome visualization produced by TaxaScope for the reference assembly (NBRC 14164$^{T}$; 6,156,701 bp). From the outermost to innermost rings, the plot displays protein-coding genes on the forward and reverse strands, positions of

tRNAs and rRNAs, GC content, GC skew, and genomic coordinates. Genes are colored according to functional categories, providing an integrated view of genome structure and compositional features.

**Table S1. Container image specifications for TaxaScope modules.**

| Module | Tool Version | Database Version* | Container Image (Release Tag) | Image Digest (SHA256, linux/amd64) |
|---|---|---|---|---|
| Prokka | 1.14.6 | v1.14.6 | docker.io/pyx07/prokka:v1-nodb-release | sha256:6b0c6e6e8b330858c9764690ead2b9d8a4160109474c2401f6eddf2347fb4e82 |
| dbCAN | 4.2.0-rc2 | db_v5_2_9-13-2025 | docker.io/pyx07/dbcan:v5-nodb-release | sha256:46e1790f026fa5a33f7d6a65c6c3c437b9122a59c2e46c62e79806db942c2dc7 |
| BUSCO | 5.8.2 | bacteria_odb12 (2025-05-14) | docker.io/pyx07/busco:v5-nodb-release | sha256:c73e3c9a4b837ed7f216fc024721593ac44290d904d1567bb9582b7984158845 |
| CheckM2 | 1.1.0 | Zenodo record 14897628 | docker.io/pyx07/checkm2:v2-nodb-release | sha256:c9a9ed455a39d26041b9a8d30edf144c02c7360190be8d06888edfb897c48134 |
| PhyloPhlAn | 3.0.67 | Zenodo record 4005620 | docker.io/pyx07/phylophlan:v3-nodb-release | sha256:8cc55c91b5a79f2df4dba8be7128e00953bb298569d1c4a4225d599f12191277 |
| antiSMASH | 8.0.4 | standalone-lite 8.0.4 databases | docker.io/pyx07/antismash:latest-nodb-release | sha256:864038983edb0c19caf45cbfcdf5bb01156da7aafb0ae73a27bbbce7e128234c |

* External; not distributed with image

**Table S2. Key libraries underpinning the visualization and graphical architecture of TaxaScope.**

| Library / Tool | Core Function | Application in TaxaScope |
|---|---|---|
| Matplotlib | Core plotting engine | Rendering phylogenetic trees, bar plots (genome size), GC content profiles, and feature distributions |
| Biopython (Bio.Phylo) | Phylogenetic tree parsing | Parsing Newick/Nexus files, computing branch coordinates, and supporting re-rooting |
| Pandas | Data integration and processing | Loading genome metadata and aligning genomic features to phylogenetic leaf nodes |
| axes_grid1 (mpl_toolkits) | Multi-axis layout | Implementing shared-axis layouts for trees and aligned metadata panels |
| CustomTkinter | GUI interaction layer | Providing interactive controls for coloring, filtering, and visualization settings |
| Pillow (PIL) | Image processing | Handling bitmap previews and lossless scaling within the GUI |
| Selenium & WebDriver | Vector rendering support | High-fidelity rendering of complex SVG/PDF reports using a headless browser |
| PyCirclize | Circular genome visualization | Generating circular plots including GC skew and gene density |

## Table S3. Quality control metrics of reference and comparative genomes used in this study.

| | | | CheckM | | BUSCO | | | | | |
|---|---|---|---|---|---|---|---|---|---|---|
| Organism Name | Strain/Isolate | Accession | Completeness | Contamination | Quality | Complete (C) | Single (S) | Duplicated (D) | Fragmented (F) | Missing (M) |
| *P. putida* | NBRC 14164$^T$ | GCF_000412675.1 | 100 | 0.41 | Excellent | 114 (98.3%) | 114 (98.3%) | 0 (0.0%) | 1 (0.9%) | 1 (0.9%) |
| *D. caeni* | DSM 24390$^T$ | GCF_000421765.1 | 100 | 0.09 | Excellent | 116 (100.0%) | 116 (100.0%) | 0 (0.0%) | 0 (0.0%) | 0 (0.0%) |
| *P. taiwanensis* | DSM 21245$^T$ | GCF_000425785.1 | 99.99 | 0.21 | Excellent | 115 (99.1%) | 115 (99.1%) | 0 (0.0%) | 0 (0.0%) | 1 (0.9%) |
| *P. monteilii* | NBRC 103158$^T$ | GCF_000621245.1 | 100 | 1.23 | Excellent | 114 (98.3%) | 113 (97.4%) | 1 (0.9%) | 1 (0.9%) | 1 (0.9%) |
| *P. plecoglossicida* | DSM 15088$^T$ | GCF_000688275.1 | 100 | 0.5 | Excellent | 114 (98.3%) | 114 (98.3%) | 0 (0.0%) | 1 (0.9%) | 1 (0.9%) |
| *P. capeferrum* | WCS358$^T$ | GCF_000731675.1 | 100 | 0.34 | Excellent | 115 (99.1%) | 115 (99.1%) | 0 (0.0%) | 0 (0.0%) | 1 (0.9%) |
| *P. alkylphenolica* | KL28$^T$ | GCF_000746525.1 | 100 | 0.56 | Excellent | 115 (99.1%) | 115 (99.1%) | 0 (0.0%) | 0 (0.0%) | 1 (0.9%) |
| *P. huaxiensis* | WCHPs060044$^T$ | GCF_003231275.1 | 99.98 | 0.56 | Excellent | 114 (98.3%) | 114 (98.3%) | 0 (0.0%) | 1 (0.9%) | 1 (0.9%) |
| *P. asiatica* | RYU5$^T$ | GCF_009932335.1 | 100 | 0.12 | Excellent | 114 (98.3%) | 114 (98.3%) | 0 (0.0%) | 1 (0.9%) | 1 (0.9%) |
| *P. shirazensis* | SWRI56$^T$ | GCF_014268785.2 | 86.82 | 0 | Good | 105 (90.5%) | 105 (90.5%) | 0 (0.0%) | 2 (1.7%) | 9 (7.8%) |
| *P. farsensis* | SWRI107$^T$ | GCF_014268805.2 | 100 | 0.15 | Excellent | 115 (99.1%) | 114 (98.3%) | 1 (0.9%) | 0 (0.0%) | 1 (0.9%) |
| *P. urmiensis* | SWRI10$^T$ | GCF_014268815.2 | 100 | 0.21 | Excellent | 115 (99.1%) | 115 (99.1%) | 0 (0.0%) | 0 (0.0%) | 1 (0.9%) |
| *P. promysalinigenes* | RW10S1$^T$ | GCF_014269025.2 | 99.99 | 0.05 | Excellent | 115 (99.1%) | 115 (99.1%) | 0 (0.0%) | 0 (0.0%) | 1 (0.9%) |
| *P. vlassakiae* | RW4S2$^T$ | GCF_014269035.2 | 100 | 0.28 | Excellent | 114 (98.3%) | 114 (98.3%) | 0 (0.0%) | 1 (0.9%) | 1 (0.9%) |
| *P. oryzicola* | RD9SR1$^T$ | GCF_014269185.2 | 100 | 0.05 | Excellent | 114 (98.3%) | 113 (97.4%) | 1 (0.9%) | 1 (0.9%) | 1 (0.9%) |
| *P. kermanshahensis* | SWRI100$^T$ | GCF_014269205.2 | 99.99 | 0.8 | Excellent | 113 (97.4%) | 113 (97.4%) | 0 (0.0%) | 1 (0.9%) | 2 (1.7%) |
| *P. anuradhapurensis* | RD8MR3$^T$ | GCF_014269225.2 | 100 | 0.02 | Excellent | 114 (98.3%) | 113 (97.4%) | 1 (0.9%) | 1 (0.9%) | 1 (0.9%) |
| *P. kurunegalensis* | RW1P2$^T$ | GCF_014269245.2 | 99.88 | 0.13 | Good | 110 (94.8%) | 109 (94.0%) | 1 (0.9%) | 1 (0.9%) | 5 (4.3%) |
| *P. faucium* | BML-PP048$^T$ | GCF_021602585.1 | 99.99 | 1.71 | Excellent | 114 (98.3%) | 114 (98.3%) | 0 (0.0%) | 1 (0.9%) | 1 (0.9%) |
| *P. fortuita* | GMI12077$^T$ | GCF_026898135.2 | 100 | 0.76 | Excellent | 114 (98.3%) | 114 (98.3%) | 0 (0.0%) | 1 (0.9%) | 1 (0.9%) |
| *P. fontis* | ID656$^T$ | GCF_028656975.1 | 100 | 0.81 | Excellent | 114 (98.3%) | 113 (97.4%) | 1 (0.9%) | 1 (0.9%) | 1 (0.9%) |
| *P. asplenii* | ATCC 23835$^T$ | GCF_900105475.1 | 100 | 0.31 | Excellent | 114 (98.3%) | 113 (97.4%) | 1 (0.9%) | 0 (0.0%) | 2 (1.7%) |
| *P. inefficax* | JV551A3$^T$ | GCF_900277125.1 | 100 | 7.35 | Excellent | 114 (98.3%) | 113 (97.4%) | 1 (0.9%) | 1 (0.9%) | 1 (0.9%) |
| *P. putida* | KCTC 1751$^T$ | GCF_024508115.1 | 100 | 0.4 | Excellent | 114 (98.3%) | 114 (98.3%) | 0 (0.0%) | 1 (0.9%) | 1 (0.9%) |

**Table S4. Comparison of TaxaScope with cloud-based platforms and conventional command-line workflows from the perspective of usability and workflow accessibility.**

| Aspect | Cloud-based platforms | CLI workflows | TaxaScope |
|---|---|---|---|
| **Target users** | General bioinformatics users | Users familiar with Linux and command-line tools | Bacterial taxonomists and wet-lab researchers |
| **Data handling** | Requires data upload to remote servers | Local execution | Local execution |
| **Setup complexity** | Moderate | High; requires software installation and dependency management | Low; one-click environment setup and automated container image retrieval |
| **Workflow integration** | Broad multi-purpose platforms | Manual workflow construction | Integrated taxonomy-oriented workflow |
| **Learning burden** | Moderate to high | High | Lower |
| **Main advantage** | Large-scale online infrastructure | High flexibility | Improved usability, accessibility, and standardized execution for genome-based bacterial taxonomy |
| **Main limitation** | Upload time, queues, non-specific interfaces | Dependency conflicts and Linux expertise required | Not intended for large-scale metagenomic community analysis |
